# More Negative Emodiversity Is Associated With Worse Mental Illness During (but Not Before) COVID-19

**DOI:** 10.1093/geroni/igab046.066

**Published:** 2021-12-17

**Authors:** Emily Urban-Wojcik, Alexandra Barnes, Dan Fitch, Andrew Kirvin-Quamme, Elizabeth Nord, Lauren Gresham, Richard Davidson, Stacey Schaefer

**Affiliations:** 1 University of Wisconsin--Madison, Madison, Wisconsin, United States; 2 Center for Healthy Minds, University of Wisconsin--Madison, Madison, Wisconsin, United States; 3 Center For Healthy Minds, University Of Wisconsin--Madison, Madison, Wisconsin, United States; 4 University of Wisconsin-Madison, Madison, Wisconsin, United States

## Abstract

Relations between negative emodiversity (NED; the variety and relative abundance of negative emotions) with depression and anxiety were examined before and during the COVID-19 pandemic. Forty-five individuals (ages 25-65) participated in two ecological momentary assessments (EMA): pre-pandemic and during-pandemic (Fall, 2020). Participants reported how much they felt 6 negative emotions several times each day for 10 days (resulting up to 91 EMA “events”). Each event’s NED was computed and then averaged using an adaptation of Shannon’s entropy. Participants with higher levels of average NED had higher levels of concurrent depression and anxiety. When adjusting for average levels of negative emotion and other covariates, NED was a significant predictor of depression and anxiety only during the pandemic. These findings, which did not vary by age, suggest that having more diverse negative emotions on a moment-to-moment basis may hold greater significance for mental illness outcomes during times of extreme chronic stress.

